# School Milk Programs in Latin America and the Caribbean

**DOI:** 10.1016/j.cdnut.2024.104541

**Published:** 2024-12-28

**Authors:** Ayala Wineman, Maria Martinez, Nicole Jacquet, Eth Ludmilla Rodrigues, Arlene Mitchell

**Affiliations:** 1 Global Child Nutrition Foundation, Seattle, Washington, United States; 2 Department of Agriculture, Food, and Resource Economics, Michigan State University, East Lansing, Michigan, United States

**Keywords:** agricultural development, children’s nutrition, dairy, Latin America and the Caribbean, school feeding

## Abstract

**Background:**

School milk programs have a long history in Latin America and the Caribbean. In recent decades, the region has undergone a nutrition transition characterized by a rise in children’s overweight/obesity, which adds new challenges for school-based programs.

**Objectives:**

This article aimed to unpack how school milk programs in the region have fared as of 2022 and what drives their success (or lack thereof).

**Methods:**

Data from the Global Survey of School Meal Programs were analyzed descriptively to broadly summarize the current state of school feeding programs in Latin America and the Caribbean. To probe the status of school milk programs, key informant interviews were conducted with school milk stakeholders in 7 countries (Brazil, Chile, Colombia, the Dominican Republic, Ecuador, Guatemala, and Honduras), and these interviews were analyzed for thematic elements.

**Results:**

Five countries were found to have operational school milk programs, whereas the programs in Guatemala and Honduras have not (thus far) endured. Programs often prioritized and took pride in local milk procurement, although there was sometimes incongruity between objectives to improve children’s diets and stimulate local agricultural development. Program implementers grapple with the taste, texture, and safety of milk, and it can be challenging to reconcile concerns over child obesity and the need to ensure the milk is appealing and accepted.

**Conclusions:**

In conclusion, programs seek creatively to overcome the aformentioned challenges wherever there is fiscal capacity and political will.

## Introduction

School meal programs—through which students are provided with meals, snacks, or take-home rations—are found throughout the world, together comprising one of the largest social safety nets [1]. Such programs are intended to address multiple cross-sectoral objectives, including, inter alia, improving children’s school attendance and performance, nutrition, and health; influencing their dietary choices; and strengthening the local agricultural economy. Worldwide, the aggregate number of children fed through school meal programs increased by ∼7% from 2018 to 2021; such growth was most evident in low-income and lower middle-income countries [2].

About two-thirds of school meal programs include milk or other dairy products, with some programs focused solely on milk/dairy and others incorporating it within a wider menu [2]. In this article, both are referred to as school milk programs. Milk and dairy products are a good source of protein and micronutrients, such as calcium, phosphorus, potassium, iodine, and vitamins A, B-2, B-12, and D, and milk can also be an important source of calories [3]. Milk or dairy consumption is associated with children’s growth and lean body mass [4]; greater micronutrient sufficiency [5]; and reduced likelihood of coronary artery disease, stroke, and heart failure (specifically when consumed in an optimal amount) [6]. The provision of milk in schools is also pursued with the aim of fostering rural development and growing a country’s dairy industry [2].

Latin America and the Caribbean (LAC) have a long history with school meal programs, such that almost every country has a national program. Although Uruguay celebrated its 100th year of school meals in 2020, and Chile’s program began in the middle of the twentieth century, countries such as the Dominican Republic, Ecuador, El Salvador, Guatemala, Haiti, Honduras, Nicaragua, and Panama established their national programs between 1995 and 2000 [7]. Many programs in LAC include (or at least purportedly intend to include) milk and other dairy products.

Over the past several decades, the LAC region has undergone a range of economic, demographic, and cultural transformations that have altogether reshaped its food system. Policy liberalization, rising incomes, improvements in infrastructure that facilitate private transport, urbanization, a trend toward rural nonfarm employment, and increases in women’s employment [8,9] have together facilitated the “nutrition transition,” a population-level dietary shift away from traditional foods with minimal processing toward higher value products, such as dairy and other animal products, and highly processed foods with high amounts of sugar, unhealthy fats, and salt [8,10,11]. This shift occurs as food is increasingly consumed away from home, often as a snack rather than a structured meal, and procured from convenience stores, fast-food restaurants, and street stalls. It also occurs alongside a shift toward more sedentary lifestyles [10].

The nutrition transition is characterized by a rise in overweight/obesity. However, because these drivers of change have affected the population unequally, all 3 types of malnutrition are present in LAC, including undernutrition and stunting; overweight/obesity and diet-related noncommunicable diseases, such as type-2 diabetes and heart disease; and micronutrient malnutrition, such as iron or iodine deficiencies [[8], [9], [10]].

Although LAC has experienced a significant reduction in the number of children under 5 experiencing stunting, which fell from 13.9 million in 1990 to 6.1 million in 2015, 11.6% of its children still experience chronic malnutrition [7]. At the same time, the prevalence of overweight in children under 5 increased from 6.2% in 1990 to 7.5% in 2019, and it has tripled in children and adolescents aged 5 to 19 y over this same interval [12].

This situation poses a serious challenge for LAC governments, which must design programs and policies that simultaneously tackle both undernutrition and the rising obesity epidemic [8]. Countries in the region have actively sought to reduce the consumption of unhealthy foods and mitigate the negative impacts of diet change. They have mandated package labels, applied taxes on sugary beverages and other unhealthy foods, and restricted food marketing targeted at children [9,10]. Interventions focused on the school food environment have potential to address multiple types of malnutrition and reverse childhood obesity trends [13,14]. Among their many functions, school meal programs can introduce children to healthy food and beverage options and convey nutrition messages with the goal of influencing their lifelong food choices [7].

This article aimed to understand how school milk programs in LAC have fared as of 2022/2023, particularly in light of the nutrition transition. We focused on milk for several reasons: policymakers and school feeding stakeholders have expressed and have exhibited a strong desire to include milk in school-based food programs, and our aim was to generate knowledge that will help these programs succeed at their goals. In addition, various milk-specific considerations need to be taken into account when designing school feeding programs. Dairy tends to be limitedly consumed at low-income levels but increasingly consumed as incomes rise; the dairy value chain has a unique structure; and the perishability of milk makes it somewhat unique among possible school menu items. The topic of milk in school-based food programs therefore warrants a “deep dive” to understand the programs’ operations and challenges.

Previous studies have broadly considered the state of school feeding in the LAC region, highlighting the importance of school meals to children’s well-being and education outcomes [15]. Studies have also focused on dairy development and nutrition in low-income and middle-income countries [16], exhorting researchers to give more attention to the most effective means of promoting dairy consumption in these countries. However, to our knowledge, no other study has focused in-depth on school milk in the LAC region. We aimed to fill this gap with a cross-country study that draws on the voices of stakeholders directly. To set this work in context, we first summarized the current scope of school-based food programs in LAC region, drawing on the most up-to-date information gathered in the Global Survey of School Meal Programs. We then focused on 7 countries that represent a wide cross-section of income levels and cultures, namely Brazil, Chile, Colombia, Dominican Republic, Ecuador, Guatemala, and Honduras. The contents of key informant interviews with stakeholders in each country were analyzed to understand whether and how school milk programs operate; the sources of milk and the nature of relevant dairy supply chains; challenges associated with the provision of milk/dairy in each context; and how the programs have responded to these challenges.

## Methods

To first measure the scale and coverage of school meal programs in LAC, we analyzed data from the 2019 and 2021 rounds of the Global Survey of School Meal Programs [2,17]. This survey is administered by the Global Child Nutrition Foundation and solicits detailed information from national governments on all large-scale school meal programs worldwide. The 2019 wave of this survey was reviewed by the University of Washington Institutional Review Board and was deemed to be exempt, as this data collection exercise did not constitute human subject research. In each survey round, an invitation to participate in the survey was extended to all national governments, with a request that each government designate a “focal point,” an individual who is knowledgeable about school feeding activities in the country and/or could gather needed information from other sources to complete the survey, and who could obtain government clearance for the results to be included in a global database. The survey was administered in English, Spanish, and Portuguese, among other languages. In total, 155 countries (including 24 of the 33 countries in LAC) participated in ≥1 round of the survey. The survey data were analyzed descriptively.

To understand the current state of school milk programs in the 7 focus countries, we then conducted semistructured interviews with key informants representing various stakeholder groups. These included representatives of government, program managers/implementers, local academics/experts on children’s nutrition or the particular school meal program, representatives of the private sector (e.g. dairy processors or school meal caterers), representatives of civil society (e.g. advocates for children’s nutrition or government transparency), and development partners. We aimed for diversity of representation among key informants because these various stakeholder categories are likely to possess different stocks of knowledge and hold different perspectives on the school milk program. For example, although government representatives are likely aware of the inner workings of the program, civil society representatives are expected to be more in tune with beneficiary experiences. Invitations were extended to potential key informants based on the authors’ desk review of each program and familiarity with the local context, and snowball sampling was also implemented whenever an informant recommended another source.

The interviews explored the extent to which milk and other dairy products are served in schools, the nature of the products and the supply chains, challenges experienced, and overall perspectives regarding the programs’ success. A list of guiding questions for the semistructured interviews is presented in Table 1.

**TABLE 1:** Guiding questions addressed in the semistructured interviews.

To what extent are milk and dairy products served in the country/school meal program? - Capture quantity by product, frequency, number and share of students reached, and school levels reached. - Is the milk/dairy considered part of a larger school meal program or a separate milk program?
What is the history of the milk program? - When did it begin? - Who were the main advocates? - What are the objectives of the program?
What is the nature of the milk/dairy products provided? - From what animal is the milk/dairy sourced? - In what form is the dairy used? (i.e. as fresh milk, yogurt, cheese, or as an ingredient in 1 or more product) - What is the milk/dairy product or ingredient used? (i.e. whole, skim, partly skim, or other) - Is the milk/product fortified? Sweetened? Salted? Flavored? - Does the product require refrigeration? - If milk is used as a beverage, is it raw, pasteurized, and/or UHT? - Where is it provided? (i.e. in school, as take-home rations)
What are the sources of the milk/dairy products? - What share of the milk/dairy used in the schools is purchased in country? - What share of the milk/dairy is procured from outside the country, and from which country/countries? - If the source is one or more business entity, is the headquarters domestic or international? What is its size? (i.e. local, national or nearly national in scale, or multinational in scale) - If sourced directly from farmers (i.e. domestic dairy farms), what is the typical farm size? - If some is purchased in country, what is the estimated proximity to the schools where it is served? - For imported products, in what form is it imported?
What does the supply chain look like? - Capture a description of the nodes, i.e. the site where the product changes hands or form between the dairy farm or the port and the children at school. - What path does the milk travel? What happens to it along the way? Who is involved?
What are the challenges associated with the provision of milk? - Note any supply chain breaks, cost overruns, food safety concerns, health concerns, taste preferences, corruption, etc.
Is the school milk program readily able to access supply to meet the demand? - How often does the program face difficulties? How widespread? - What are the reasons for the difficulty? (Responses may include lack of supply; quality issues with the supply; lack of funding/payments for suppliers; price increases; issues with transport or storage; or other) - What happens when there is not enough supply? (i.e. is there a product that schools substitute for the milk/dairy product, or do schools go without any dairy product? Are some school levels prioritized when supply is limited?)
What food safety measures are taken to ensure the safety of milk/milk products? - Answers may include factory controls, quality control checks at the school level, or use of bottled water for preparation of powdered milk, among others.
How is the school milk/dairy program funded? - This question relates partly to whether it is considered a separate program from the main school meal program. - Is the funding considered secure or unpredictable?
Who implements the program and how is it implemented? - The answer may include schools, communities/local governments, national governments, private companies, etc. Multiple entities may have complementary responsibilities. - Which cost model is used? (e.g. milk may be provided at full cost, at a subsidized cost, and free of charge). - On what basis are children targeted to receive the milk?
Is the school milk/dairy program well liked? - Are schools, communities, families, and students supportive of the inclusion of milk/dairy? Why or why not? - Are schools, communities, families, and students satisfied with how the program is implemented in practice? - Has government been supportive of the inclusion of milk/dairy products? What can be said about “political will” to support the milk program? - Is there an intention to scale up (or down) the milk/dairy program in the future?

Interviews were conducted between November 2022 and March 2023. In most cases, they were conducted virtually (over Zoom video communications), although 2 interviews in Colombia were held in person. The languages used were Spanish for the 6 Spanish-speaking countries and Portuguese for Brazil. A statement of informed consent was read aloud, and verbal consent was secured before initiating each interview. In total, 18 interviews with 21 individual stakeholders were conducted. These included representatives of government (8), the private sector (11), academia (1), and a nongovernmental organization (1). A list of informants is found in Supplemental Table 1.

The interviews were recorded, and while 1 member of our author team was present at each interview, a second member with appropriate language skills also listened to the recording. The 2 then worked together to produce detailed notes (in English) on what was discussed, and the notes from the 18 interviews were assembled for analysis. In a series of team meetings, we discussed the key takeaways from each section of the interview writeup. The outcome of this step was a country-by-country summary of the state of the school milk program in each country, focusing on whether and how programs operate; the nature of supply chains; and challenges experienced in the provision of milk/dairy. These results are contextualized with reference to a nonsystematic literature review on the topic of school milk programs in each country.

In the next step, we reviewed the interview contents with the aim of identifying cross-country themes. Toward this end, we assigned preliminary codes to the content of each interview, and in an iterative manner, we then identified commonalities among the initial set of codes and grouped them into larger organizing themes [18]. The final set of themes was determined by consensus.

## Results

### School Meal Programs in LAC

**FIGURE 1:**
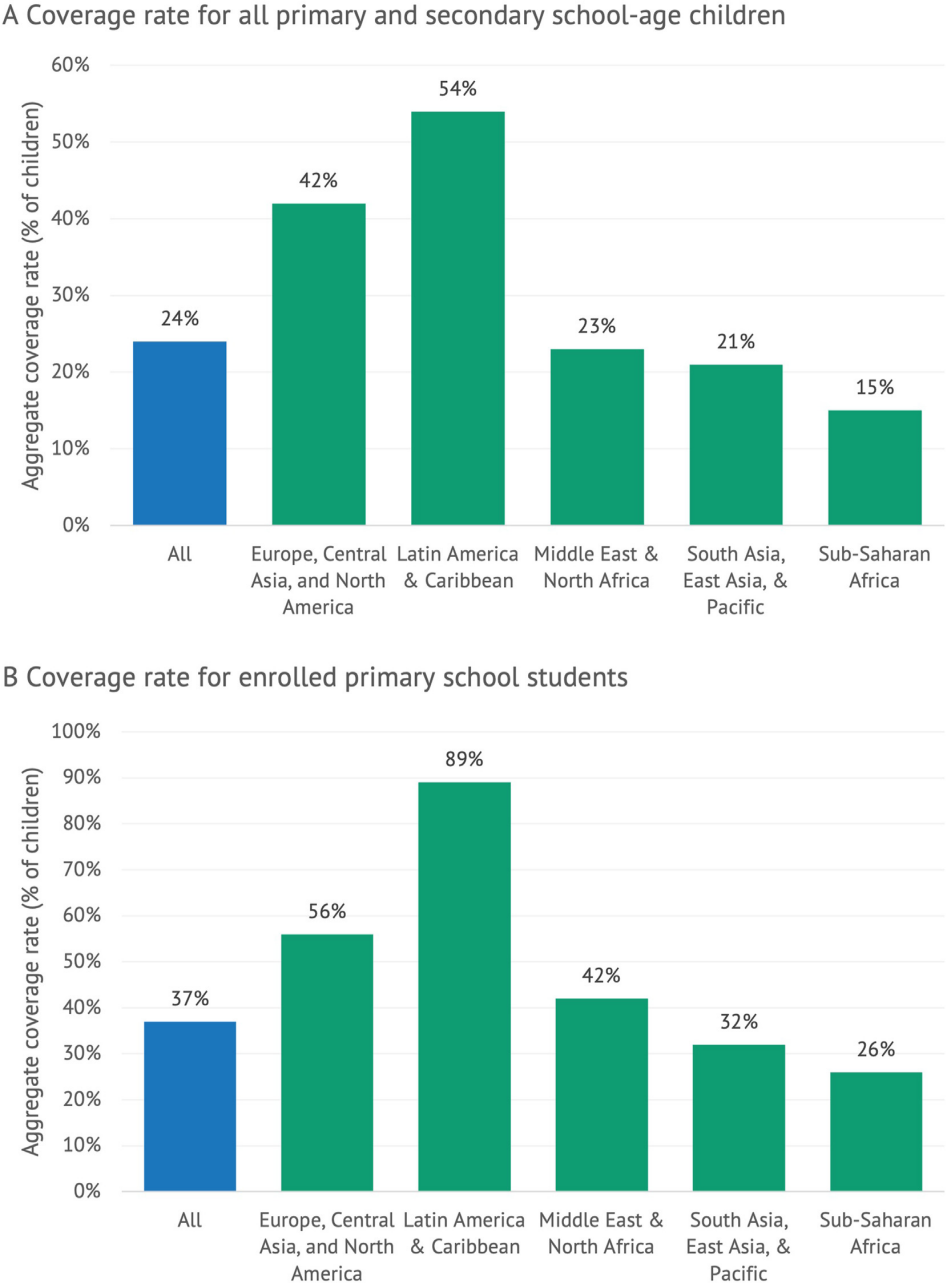
School feeding coverage rates across regions of the world. (A) Coverage rate for all primary and secondary school age children. (B) Coverage rate for enrolled primary school students. Source: Global Survey of School Meal Programs [2,17]. Note: The data used in the aforementioned analysis refer to the most recent survey round of the Global Survey of School Meal Programs in which a given country participated. This reflects the 2021 round for 140 countries and the 2019 round for an additional 15 countries. The reference year in the 2021 round was the school year that began in 2020, although the reference year in the 2019 round was the most recently completed school year as of the time of data collection (the year that began in 2017 or 2018, depending on the school calendar in each country). The 155 countries in the Global Survey of School Meal Programs database together hold 87% of the world’s population. The region groupings used in this figure loosely match those employed by the World Bank. However, South Asia is combined with the East Asia/Pacific region, and North America is combined with the Europe/Central Asia region as there are just 2 North American countries.

Results from the Global Survey of School Meal Programs with data for 24 LAC countries—which together hold 87% of the region’s population—reveal that the LAC region stands out for its achievements related to school feeding. For example, over half (54%) of all primary and secondary school age children in the LAC region benefit from school feeding programs (Figure 1A) [2,17]. This value is higher than any other region in the world. Because school meal programs target the primary school level more often and more intensely than other school levels, and because there are fewer out-of-school children among younger ages, the school feeding coverage rate is higher when it is calculated narrowly with respect to the population of enrolled primary school students. Using this alternate construction of the coverage rate (as has recently been proposed as a new Sustainable Development Goal indicator [19]), the school feeding coverage rate in LAC is an astounding 89%, far higher than the worldwide rate of 37% and higher than any other region (Figure 1B). In total, 68.4 million children in the LAC region (among the countries included in the survey database) receive some food through their schools.

The number of children reached by large-scale school meal programs in each focus country is presented in Table 2 [2,20]. Among this set of countries, Brazil’s school meal program is largest, reaching over 40 million students in preschool, primary school, and secondary school. The school age coverage rate is defined as the share of children of primary or secondary school age in each country that receive or procure some food through school meal programs. Brazil has the highest school age coverage rate, reaching 92% of school age children, although Guatemala has the lowest at 41%. Because many school meal programs target mostly the primary school level, column 3 of Table 2 breaks out the coverage rate for primary school age children. Ninety percentage of primary school age children in Honduras benefit from school meal programs.

**TABLE 2:** School feeding reach and coverage rate by country (2020 school year).

Country	No. of children fed (all grade levels)	Primary and secondary school age coverage rate (%)	Primary school age coverage rate (%)
Brazil	40,189,426	92	Unknown
Chile	2,029,882	68	Unknown
Colombia	4,825,218	48	52
Dominican Republic	1,743,395	65	75
Ecuador	2,941,952	62	78
Guatemala	2,526,650	41	62
Honduras	1,256,227	65	90

The aggregate budget for school meal programs in the LAC region is United States dollars (USD) 4.23 billion (across the 24 countries in the survey database), with an average investment per student beneficiary of USD 62 per year. (The values of USD 4.23 billion and USD 62 are calculated using the average exchange rate between the local currency and the USD over the course of the relevant school year for each responding country.) However, when accounting for differences in purchasing power parity, the average investment in the LAC region is 148 international dollars (I$) per year per student beneficiary. Although 69% of countries globally include school feeding as a line item in their national budget, this value is 96% in the LAC region—again underscoring the region’s exceptional commitment to school feeding. The LAC region also stands out when it comes to linkages between school meal programs and agricultural development, with 83% of programs engaging with farmers. For example, in Brazil, 100% of food for the school meal program is procured domestically, with a minimum of 30% purchased from small-scale family farmers. In Chile, some school food must be purchased from small-scale family farmers or other local producers, with specific requirements varying across different territories.

A large share (83%) of school meal programs in LAC report that they include milk or other dairy products. This coincides with the growing role of dairy in LAC. Specifically, whole milk equivalent consumption per capita increased in LAC from 80 kg in 1965 to 125 kg in 2015 [9], and over the past 30 years, the diet of most Chileans has shifted from one lacking in animal protein and calcium to one that is particularly high in meat and dairy products [11]. Overall, the survey results confirm that school meal programs are sizable in LAC and suggest that milk features prominently (or is at least intended to feature prominently) in many programs.

### School Milk Programs in LAC

A summary of information gathered through key informant interviews in each of the 7 countries is provided in Table 3 [2]. Active school milk programs were documented in 5 countries; specifically, the milk programs seem to be inactive or not actualized in Guatemala and Honduras. The milk and other dairy products are sourced entirely or almost entirely from within the country in 4 cases, whereas the Dominican Republic imports most of the dairy for their program. The various programs exhibit a wide mix of approaches to management: In Guatemala, decisions regarding the school menu are made at the school level, although in Brazil, these decisions lie at the municipality level. Some programs are evidently vulnerable to global supply chain disruptions; this is the case in the Dominican Republic, which imports much of its milk, and also Colombia, which sources its milk domestically but imports inputs for milk production. Notable observations from the key stakeholder interviews in each country are detailed below.

**TABLE 3:** Highlights of country case studies.

	Brazil	Chile	Colombia	Dominican Republic	Ecuador	Guatemala	Honduras
School milk program active as of 2023	Yes	Yes	Yes	Yes	Yes	No	No^1^
No. of children that receive milk/dairy through their schools	∼40 million	1.6–2.4 million	∼4 million	∼1.3 million	∼2.9 million	NA	Unknown
Fresh, UHT-treated, or powdered milk	Fresh, UHT, powder	Fresh, UHT, powder	Fresh, UHT	Fresh, UHT, powder	UHT	NA	NA
Full-fat, partly, or fully skimmed milk	—2	Whole or partly skimmed	Whole	Whole	Whole	NA	NA
Fortified milk	No^3^	No	Yes	Yes	Yes	NA	NA
Flavors or sugars added to milk	Yes	Yes	Yes	Yes	Yes	NA	NA
Other dairy products served	Yogurt	Yogurt, cheese, pudding	Yogurt, frescolanta, cream cheese	Cheese	Yogurt, quinoa-dairy drink	None noted	None
Dairy sourced from within the country	All	Most	All	Very little	All	NA	NA
Small-scale/family farmers given preference	Yes	Yes	No	—	Yes	Yes	Yes
Animal source of dairy	Cow	Cow	Cow, buffalo	Cow	Cow	NA	NA
Centralized or decentralized program management	Decentralized to municipality level	Zone-level centralization	Decentralized to ETC level	Centralized	Territory-level centralization	Decentralized to school level	Mixed model
Key successes	Strong engagement with local farmers	Strong support from schools and communities	Strong public support	Some incentives provided to local dairy farmers	Reverse auction effectively used to identify suppliers	PAE requires the school food basket to be locally sourced	By law, milk is to be served every school day
Key challenges	Within-municipality sourcing requirement is difficult to meet	Children’s limited receptivity to skimmed or fortified milk	Standards difficult for small producers/processors to meet	Vulnerable to global supply shocks	Government offers low prices to milk/snack suppliers	Because milk is expensive, school-level committees do not purchase it	Milk program has not been funded

ETC, Centralized Territorial Entity for Education; NA, not applicable; UHT, ultraheat treatment/ultrahigh temperature.

1 It seems milk or other dairy products are not currently served in schools through the national school meal program of Honduras, although the World Food Program does provide powdered milk and dairy in a small program operating in the most food insecure regions.

2 Dashes indicate information not gathered.

3 Information derived from the responses to the 2021 Global Survey of School Meal Programs [2].

#### Brazil

The National School Feeding Program (Programa Nacional da Alimentação Escolar [PNAE]) of Brazil dates to 1955. Although it initially functioned in a centralized manner, management was devolved in 1994 to Brazil’s many local municipalities, which are entirely responsible for the purchase and provision of food. The program places a heavy emphasis on local food procurement, with ≥30% coming from family farms [21]. In this regard, it is viewed as a model for many countries [22]. The PNAE is funded by the federal government and serves over 40 million children [23].

Cow milk is frequently served in schools, as its consumption is routine in the Brazilian diet. The milk is of national origin and is often produced by local industries and/or family farmer organizations. According to key informants, foods procured from family farms can include products that have been industrially processed (e.g. yogurt), as long as the raw product originated on a family farm. There does not seem to be a detailed breakdown of the share of milk/dairy in the PNAE that comes from family farms or other sources.

Brazil is the largest producer of milk in LAC and is home to the world’s second largest dairy herd [24]. At least some milk is produced in 554 of Brazil’s 558 microregions [25]; however, production is not distributed uniformly, and the state of Minas Gerais accounts for 25% of national production, although 3 southern states (Paraná, Santa Catarina, and Rio Grande do Sul) together account for 39% of national production [26]. Production systems in Brazil are diverse, spanning small producers with no equipment to specialized producers that use advanced technology and produce over 60 L/d/cow [25]. Dairy producers generally organize themselves into associations or cooperatives for marketing purposes, with few family farmers’ cooperatives capable of processing milk themselves.

According to the key informants, the PNAE has no difficulty in accessing milk supply. However, various requirements in the program present challenges. For example, there is a territorial requirement that family farm production should be from a farm located within the municipality, and sometimes there is not enough production of a given product within a given municipality. Family farmers also have difficulty meeting sanitary standards, especially for animal-origin products [27]. Milk served in the PNAE must be pasteurized and must comply with sanitary regulations, and powdered milk may only be served in exceptional circumstances [28]. According to EMBRAPA (2022), Brazil’s dairy sector is characterized by lower average productivity (2192 L/cow/y) than neighboring Argentina (7400 L/cow/y).

#### Chile

Chile’s Programa de Alimentacion Escolar (PAE) was introduced in 1964 and is implemented by the National Board of School Aid and Scholarships (Junta Nacional de Auxilio Escolar y Becas) within the Ministry of Education. The PAE provides food daily (breakfast/snack and lunch) to vulnerable students at all school levels. The Ministry of Education contracts private companies to handle the supply, preparation, and distribution of food, with bidding competitions held among catering companies to supply schools in each of 3 zones in the country. Estimates of the number of children that receive some food through the PAE range from 2.03 million [29] to 2.40 million [2].

The PAE has been providing schoolchildren with milk since 1980. Milk is distributed every school day; yogurt is provided once per week; cheese is sometimes included in sandwiches; and dairy-based desserts, such as mousse and rice pudding, are also served. Milk products distributed through the PAE are made of pasteurized and/or ultraheat treatment (UHT) cow milk. The milk is alternately whole or partly skimmed and is sometimes sweetened with artificial sweeteners or flavors (e.g. chocolate and vanilla), and we heard that fully skimmed milk is regarded as tasteless by the children. Although the PAE has experimented with serving fortified milk, students were not receptive, and the program is looking to develop another fortified formula that will be more acceptable.

As Chile stretches over thousands of kilometers, powdered milk has generally been easier to transport. However, fresh milk is preferred to reconstituted (powdered) milk in terms of taste and texture, and powdered milk, which must be prepared in schools, is sometimes deemed too diluted, too concentrated, or poorly mixed (i.e. lumpy). Nevertheless, fresh milk is much more expensive, and the PAE, which has served fresh milk for some time, is now resuming use of some powdered milk.

According to key informants, the milk distributed through the PAE is mainly produced within Chile. To ensure adequate supply, the PAE also purchases from neighboring countries, with sources of imports (including Argentina, Brazil, and Uruguay) differing across the north, center, and south of the country. However, because trade disruptions sometimes occur, the PAE prefers to rely on domestic supplies to best ensure the product’s availability and safety. The quality of milk processing and distribution in Chile is regarded as high, and the country has several plants that produce UHT milk.

Schools, communities, families, and students in Chile are highly supportive of the inclusion of milk/dairy in the PAE. A “Thanks To Milk” program, organized by the Dairy Consortium, raises awareness of the vulnerability of PAE beneficiaries and advocates for retaining milk in school meals. Political will to improve children’s nutrition is high, and the government increasingly aims to augment the consumption of animal protein among preschool-aged and school-aged children.

#### Colombia

The School Meals Program (PAE) in Colombia began in 1936. In 2015, the government decentralized the administration of the PAE to the 97 Centralized Territorial Entities for Education (ETCs), making them completely autonomous and responsible for managing the contracting, execution, and supervision of the program. The PAE is implemented in primary and secondary public schools, reaching ∼4 million children; it does not serve the 3 million children in the country who are enrolled in private schools.

Milk is widely consumed in Colombia, with the exception of indigenous communities where we heard that efforts are made to gradually introduce dairy into the local diet. Milk and dairy products are provided in the PAE and are distributed in the school lunches (prepared onsite) as well as the complementary industrially prepared meals/snacks (prepared offsite). Dairy products on the school menu include fresh whole milk; UHT whole milk; powdered milk; condensed milk; frescolanta (made of whole milk, sugar, whole milk powder, and flavors such as vanilla or strawberry); plain or flavored fortified yogurt; cream cheese; and arequipe (sweet caramel sauce made with sugar and milk). According to key informants, only cow milk is served to young children; however, for older children, buffalo milk is also used in yogurt and cheese. The milk is fortified with iron and only whole (not skimmed) milk is provided.

The milk and dairy products used in the PAE are entirely produced in Colombia, with powdered milk used in the more remote regions, such as San Felipe and Guainía. As one key informant noted, “A school milk program should be linked to the development of the country and help make the national dairy sector more competitive.” We spoke with a representative of the Cooperativa Lechera de Antioquia (COLANTA), a cooperative with over 10,000 farmer-members, which provides milk and dairy products for the PAE in Antioquia (Medellín) and Bogotá. COLANTA collects the milk directly from the farms and purchases 100% of the milk produced by its associates. If it happens that production from its associates is not sufficient, COLANTA purchases milk from other Colombian dairy farmers or limits its production to its most in-demand products.

According to the Colombian Association of Milk Processors (Asociación Colombiana de Procesadores de la Leche), 1 source of tension in the PAE is that the standards for suppliers bidding on government contracts for the program are too complex or stringent to be met by small-scale and medium-scale actors. For this reason, we heard that the provision of milk and dairy products to the PAE is concentrated in the hands of just a few large suppliers. Another challenge is high fuel prices, which raise the cost of transporting milk from farm to processing plant and on to consumers. Key informants also reported some friction between the priorities of affordability and food safety, with the latter requiring investments that raise production costs.

#### Dominican Republic

School feeding began in the Dominican Republic in 1943 and was institutionalized as the PAE in 1979. The PAE is led by the National Institute of Student Welfare (Instituto Nacional de Bienestar Estudiantil [INABIE]), which is attached to the Ministry of Education [20]. Milk has been distributed to schoolchildren in the Dominican Republic for over 20 y, with an estimated 1.3 million children receiving some milk through their schools [29]. Fresh milk is served for breakfast and as a snack each school day, 5 d/wk, and the milk is distributed free of charge.

We heard that the government allocates ∼USD 96 million per year for the dairy component of the PAE, amounting to USD 0.28 per 200 mL dairy fluid ration. The program is wholly financed by the government, and INABIE issues tenders to private companies for the distribution of dairy products. In addition to directly funding the milk program, key informants indicated that the government also provides at least some incentives to dairy farmers to promote local milk production.

Most schools in urban and periurban areas (comprising ∼60% of schools) serve UHT milk, and urban schools with electricity (∼25%) serve fresh milk. Meanwhile, rural schools in especially hot climates, comprising 15% of the schools, serve powdered milk. As we heard, this is because the UHT packaging seals can give way at temperatures above 35°C, a common temperature along the desert-like frontier with Haiti. In addition, milk powder is easier to transport in areas where truck access is limited. In these remote areas, fortified milk powder is diluted directly at the school, and the children receive 400 mL of plain or chocolate-flavored milk each day. However, according to key informants, if lumps form during dilution, the milk may become noncompliant with food standards. The milk served in the PAE is whole and fortified. For sweetened milk, ∼13 g of cane sugar are added per liter, and even more is added to chocolate-flavored milk to overcome the bitter taste of cocoa. In rural schools, dairy is also distributed as cheese, and milk is sometimes included as an ingredient in other dishes.

Although the Dominican Republic has several UHT plants, pasteurizers, and dairy powder blenders, it lacks dairy production capacity relative to its needs, and much of its milk is imported. The European Union dominates as a source of powdered milk imports. According to key informants, this is because Europe provided food assistance to the Dominican Republic in the 1960s, and people grew accustomed to the flavor profile of European milk.

#### Ecuador

The PAE in Ecuador began in 2016, though the World Food Program had implemented school feeding in the country before the launch of this program. Management and funding for the PAE are now the responsibility of the Ministry of Education. Since 2016, private companies have been able to bid for contracts to supply processed products (such as UHT pasteurized milk) to the PAE, and this occurs through reverse auctions in each of 8 zones in the country. According to key informants, there is an effective system of government audits to check for quality and compliance with standards. As of 2020, the PAE provided food to over 2.9 million schoolchildren in ∼12,800 public schools [23].

Milk rations are provided to children several times per week, including UHT whole and flavored milk [30]. The PAE also serves yogurt and a milk-based drink made of cereals (especially quinoa) that must contain ≥50% milk. Rations in the PAE include 200 mL plain whole milk served 1 d/wk, 200 mL flavored whole milk served 1 d/wk, and 200 mL of a milk-based drink made of cereals served 2 d/wk. All milk distributed in schools is fortified, and the PAE does not serve powdered milk.

According to key informants, the milk provided in the PAE is entirely purchased in country. However, it is possible for some cheese, butter, and other dairy products to be purchased from abroad, especially from Colombia. It is required that 30% of the products distributed in schools must come from small producers. Small farmers in Ecuador are grouped into associations to facilitate the collection and distribution of milk; this enables them to receive a higher price than if they were to sell milk directly from their farms.

In Ecuador, surveys have revealed that a large majority of schools would like to continue receiving school food; this also includes the provision of milk. However, there are some criticisms of the PAE. For example, the government offers low prices for the school foods, and suppliers have asked for these to be raised.

#### Guatemala

Guatemala’s school meal program (PAE) was introduced in 1956, with universal coverage at the preprimary and primary levels in all public schools. An estimated 2.53 million children benefit from the PAE [23]. The menus must include a solid food, 2 beverages, and a complement as needed to meet nutritional requirements, and the PAE requires that 50% of food purchases be sourced from local family farmers. Parents’ organizations at the school level are responsible for selecting the precise menu and purchasing the food, and the government deposits funds into a bank account for each organization. We heard that the budget for the PAE has grown from USD 0.30 per child per day several years ago to USD 0.75 today. The government funds the PAE with some external support from Catholic Relief Services and the McGovern-Dole Food for Education and Child Nutrition Program [23].

From the 1970s until 1992, the government set a ceiling for milk prices, which disincentivized local milk production and processing. Many milk factories shuttered—effectively precluding Guatemala’s self-sufficiency in milk production—and dairy imports increased. Most dairy imports in Guatemala come from Central America: Specifically, we heard that about half of the UHT milk comes from Costa Rica, Nicaragua is a main source of powdered milk, and El Salvador is a main source of processed cheese. Guatemala has several UHT milk factories of its own. However, to survive in the current economic environment, many dairy processors specialize in expensive cheeses or yogurts.

In 2003, a national plan was introduced to revitalize the country’s dairy sector, and this included the distribution of milk to schoolchildren. From 2005 to 2007, the “Glass of Milk” program was implemented. It reached 600,000 children, was the responsibility of the Ministry of Agriculture, and was based on local milk purchases. The program distributed whole and unsweetened milk with an explicit aim to cultivate a preference for the plain product. As one informant noted, “It is better not to acclimate children to drink sweetened or flavored milk.” A key informant noted that, in regions of the country with a high concentration of indigenous people, the children sometimes experienced stomach aches or other discomfort when they were first served milk. After 2007, milk was replaced by a non-dairy Incaparina drink made from maize and other ingredients.

We learned from key informants that milk is currently not, in actuality, provided in the PAE. Although nutritional guidelines for the program do include milk, it seems that local milk is relatively expensive and the PAE mostly requires that milk be locally sourced. In practice, milk is therefore rarely included in the school food basket. Factors that influence the cost of local milk include cheaper imports that depress local production, sanitary/quality standards that many local farmers cannot meet, and a lack of support for dairy farmers.

According to key informants, the government feels that the Guatemalan population should consume more dairy products to reduce malnutrition; local milk producers are convinced that school milk is a good opportunity; and even importers are in favor of a school milk program, as Guatemala is potentially a large market. However, folding milk into the PAE has not been effective. Key informants feel there needs to be a standalone school milk program, like what was in place in 2005–2007, with an adequate budget and objectives that include the development of the Guatemalan dairy sector.

#### Honduras

School feeding began in Honduras in 1962, and since 2000, the PNAE has operated within the scope of the Ministry of Development and Social Inclusion and the Ministry of Education. The program has universal coverage, reaching prebasic and basic levels of education and, progressively, the intermediate level. An estimated 1.26 million children benefit from the PNAE [2]. Although the program historically had a centralized design, a mixed supply model is now being introduced with fresh vegetables and eggs purchased in a decentralized manner to complement the basic food basket [23]. Parents are responsible for preparing food and supplementing the basic ration.

In 2008 and 2009, milk was distributed in schools through a short-lived program. Under the “Vaso de Leche” (Glass of Milk) law (Decree No. 54-2010) passed in 2010, 200 mL of fresh milk is to be served to every schoolchild on each school day, summing to over 200 d/y. Unfortunately, when the law was passed, the government did not establish a dedicated line in the national budget for its implementation. In fact, the government has never released sufficient funds, and the program has not been implemented to date. It is therefore our understanding that milk and other dairy products are not currently served in schools through the PNAE. (A key informant did clarify that powdered milk and cheese are provided to schoolchildren through a small program implemented by the World Food Program in the most food insecure parts of the country. However, the use of powdered milk in schools is problematic, as the water used to dilute it is sometimes of poor quality.)

The PNAE faces challenges mostly due to an insufficient budget. Key informants noted that dairy farmers in Honduras have low productivity and experience high production costs, especially for electricity. Moreover, Guatemala and El Salvador are said to purchase a large share of local milk production. Because foreign buyers pay in advance, local dairy producers prefer to sell to them rather than to domestic industries or, potentially, to the PNAE.

According to key informants, political will to implement the school milk program in Honduras is decidedly weak. There is a perception in some quarters that the Vaso de Leche law was enacted mostly to gain political support from milk processors, resulting in less than enthusiastic support for its implementation. Going forward, it seems the PNAE is at least tentatively hoping to resume milk distribution, and commune mayors are helping the PNAE to reestablish contact with local milk producers. However, even the task of collecting contact information requires funding, which presents a challenge.

## Discussion

In this article, we broadly sought to describe the current state of school milk programs in 7 countries in the LAC region. We aimed to understand whether the programs perceive themselves (and are perceived) to be meeting their goals, what are their sources of strength, what are the challenges they face under present-day conditions, and how they seem to be approaching these challenges. What emerges is a diverse story, with clear successes in some countries and failures of capacity and follow-through in other settings. Further, we discuss several themes from this exercise.

First, programs often prioritize and take pride in local milk procurement. In several countries, the school milk program is designed to procure at least some milk from domestic producers. In Brazil, all food is sourced domestically, and 30% of purchases comes from family farmers. In Guatemala, the school meal program requires that half of food purchases be sourced from local family farmers, and the short-lived Glass of Milk program was based on local milk purchases. This emphasis on local milk procurement is noteworthy, as LAC holds 8% of the world’s population but now produces over 11% of its milk. Milk production in the region has grown by 36% from 2000 to 2018 (outpacing growth in the United States), although milk production efficiency (quantity/head of cattle) grew by 29% between 2000 and 2017 [24].

However, our interviews also reveal that there are trade-offs when a school milk program is used to support a lagging dairy sector. In Guatemala, for example, school-level committees must select the school menu with a limited budget. In this case, the high price of local milk, coupled with a requirement that milk be locally sourced, seems to preclude the provision of milk to schoolchildren. The relatively high cost of milk is at least partly a reflection of levels of dairy cattle production efficiency in LAC (at 3427 lb/head) that are far lower than the global average (5358 lb/head) [24]. These trade-offs should be acknowledged when pondering the extent to which a school milk program can serve the dual purpose of getting milk to children and stimulating agricultural development. At the same time, short-term benefits of milk imports can potentially bring long-term costs for the domestic dairy sector. In other words, there may also be a trade-off over time.

A second theme from our interviews is that the success of school milk programs is determined by both fiscal capacity and political will. Across the 7 countries of this study, there is wide divergence in gross domestic product per capita [31]. It is noteworthy that the wealthiest country, Chile, has a school milk program that operates consistently and effectively and boasts of strong public support, although Honduras and Guatemala, the 2 poorest countries in the set, do not seem to have milk provision in schools—even when it is mandated under the law. The high cost of providing (especially fresh) milk that is palatable and safe raises questions about where scarce public dollars should be allocated. Nevertheless, it is difficult to parse out the relative importance of political will compared with fiscal capacity. In Honduras, the lack of implementation was specifically attributed to a lack of political will.

A third theme is the extent to which program implementers grapple with the taste, texture, and safety of milk. Key informants from many of the countries recounted the ongoing challenge of providing milk that children find tasty and appealing. In Chile, fully skimmed milk is considered tasteless, and in the Dominican Republic, it was noted that lumps formed during preparation can render the reconstituted milk noncompliant with food standards. In Guatemala, regions with a high concentration of indigenous people were thought to be less receptive to milk because they are unaccustomed to it. In Honduras, the water that would be used to dilute milk powder in rural schools is sometimes unsafe, with potential implications for children’s health. Program implementers have been considering all variations of how milk can be provided to balance the priorities of healthiness, safety, transport costs, longevity, and acceptability among young consumers.

A fourth theme is the tension between concerns over child obesity and an imperative to ensure the milk is accepted. Rates of child overweight and obesity in these countries are alarming [8,9]. Across LAC, the prevalence of overweight in children under 5 increased from 6.2% in 1990 to 7.5% in 2019, and it has tripled in children and adolescents over this same interval [12]. It is therefore noteworthy that fully skimmed milk is not currently on the school menu in any country. Although milk already has sugar (lactose), it is sometimes sweetened or flavored in Chile, Colombia, the Dominican Republic, and Ecuador (Table 3). In Colombia, it may even be the case that only flavored milk is provided, as we heard complaints of strawberry-flavored milk being served without variation. Studies have documented the implications of students’ beverage choice on their sugar intake [32]. The provision of milk to children in Chile, with an associated instruction to refrain from drinking sugar-sweetened beverages, has been found to have beneficial impacts for children’s growth and lean body mass [4]. In the United States, students’ choice of fat-free chocolate milk has been found to result in greater calorie and sugar consumption, compared with a selection of low-fat white milk [33]. The extent to which milk served in schools in LAC may drive increased sugar consumption, both in and outside of school, merits further attention.

A fifth theme is that dairy supply chains are complex, presenting an obstacle in countries with limited infrastructure. Although fresh milk is clearly preferred, countries must consider the longevity of this product and the feasibility of its transport. The Dominican Republic, for example, allocates different types of milk in different areas based on logistical concerns: Urban schools with electricity receive fresh milk; most other schools receive shelf-stable UHT milk in high-quality packaging; and remote schools in hot climates receive powdered milk. In all countries, transporting fresh milk is expensive, and when milk needs to be imported due to limited domestic supply, it is usually (the somewhat less preferred) milk powder that is traded across borders. Per-animal milk production in LAC is well below United States and European productivity levels [24]; this represents a major challenge for a school milk program that is dependent on affordable local production. On one hand, school milk programs can be an avenue for development of the local dairy sector by providing a predictable market. On the other hand, in settings without high productivity, safe handling and storage systems, and/or efficient processing and transport facilities, a school milk program may have limited chance of succeeding.

Although school milk programs in the 7 focus countries continue to wrestle with these frictions and have not necessarily identified “win-win” solutions that completely dissolve the tension, we observed that program implementers strive earnestly and creatively for balance. Thus, implementers in Chile continue to work on a formula for fortified milk that is regarded as tasty, and the government of Colombia responded to a finding of iron deficiencies in the school age population (MINSALUD, 2015) [34] by requiring that milk in the school milk program be iron fortified. In 2017, the government of Guatemala developed a livestock strategy that includes technical assistance to local dairy producers, and we heard of a pilot project to help dairy farms improve their standards and practices so they can supply milk to nearby schools. All countries in the study demonstrate a strong desire to provide local milk to schoolchildren. Although this goal may not be within reach in all settings in the immediate term, it underscores a need to raise dairy productivity and processing capacity in LAC to levels that are internationally competitive.

We acknowledge that this study was limited in its depth by the relatively small number of key informant interviews in each country. Although the cross-country perspective afforded us a broad view of the topic across LAC, a future extension of this research may focus more narrowly on one country at a time with a greater sample size to gain a deeper understanding of the topic. The number of informants also limited our ability to draw out themes according to stakeholder category; however, an analysis of divergences in perspective would have been illuminating. Future work may also probe more deeply into topics that did not surface organically in our qualitative interviews, such as the climate change impact of dairy production/consumption [35] and stakeholder perspectives on the appropriate role of dairy in local diets.

Nevertheless, several policy implications can be identified. When planning school milk programs, it is critical to ensure adequate fiscal capacity to accomplish what is set out on paper. When setting school menus, programs should have clarity regarding their priorities around nutrition and obesity prevention. We hope this might drive programs to purposefully direct children toward healthy food habits by discouraging or prohibiting the provision of sugar-sweetened or flavored milk. When designing the system of dairy procurement, programs should consider both the benefits and costs of leveraging the program to support the domestic dairy sector. Procurement policies that alternately rely on domestic/local supply or draw on imports likely come with different cost and coverage implications, as well as implications for the country’s longer term economic health. Rather than assume any one procurement method is superior to others, we encourage programs to base their decisions on rigorous benefit-cost analyses of various procurement options. A final lesson from this study is that programs can learn a great deal from one another, and we hope they glean insight from others’ experiences—both positive and negative—to better attain their own goals.

## Author contributions

The authors’ responsibilities were as follows – AW, AM: designed the research; ELDGVNR, MM, NJ: conducted research; AW, ELDGVNR, MM, NJ: analyzed the data; AW: wrote the paper; AM: had primary responsibility for final content; and all authors: read and approved the final manuscript.

## Data availability

Data described in the manuscript will be made publicly and freely available without restriction at https://gcnf.org/global-survey. Analytic code will be made available upon request at info@gcnf.org.

## Funding

This study was jointly funded by the Global Child Nutrition Foundation (GCNF) and the United States Dairy Export Council (USDEC) under a Memorandum of Agreement. USDEC had no involvement in the study design; collection, analysis, and interpretation of data; or writing of the paper; and has placed no restrictions regarding publication.

## Conflict of interest

AM reports financial support was provided by United States Dairy Export Council. The other authors report no competing interests.
