# Supplementary material for: School Milk Programs in Latin America and the Caribbean
Source: Curr Dev Nutr. 2024 Dec 28;9(2):104541. doi: 10.1016/j.cdnut.2024.104541 (PMC11795587; doi:10.1016/j.cdnut.2024.104541)
Supplement: multimedia component 1 [file mmc1.docx]

**Supplementary Material**

**Supplementary Table 1: Summary of Outreach to Key informants**

| **Country/Region** | **Agency/Affiliation** | **Stakeholder group** |
| --- | --- | --- |
| Brazil | Companhia Nacional de Abastecimento (National Supply Company) | Government |
| Brazil | Ministry of Education | Government |
| Chile | Federación Gremial Nacional de Productores de Leche (FEDELECHE) | Private sector |
| Chile | Exporlac (Association of Exporters of Dairy Products) | Private sector |
| Chile | Department of Student Nutrition, JUNAEB (National Board of School Aid and Scholarships) | Government |
| Chile | Dairy Consortium | Academia/Research |
| Colombia | Ministry of National Education - Unidad Administrativa Especial de Alimentación Escolar – Alimentos Para Aprender (UAPA) | Government |
| Colombia | COLANTA (Cooperativa Lechera de Antioquia) | Private sector |
| Colombia | ASOLECHE (Colombian Association of Milk Processors) | Private sector |
| Dominican Republic | PROAGRO Dominicana | Private sector |
| Dominican Republic | PROAGRO Dominicana | Private sector |
| Dominican Republic | PROAGRO Dominicana | Private sector |
| Ecuador | Ministry of Education | Government |
| Ecuador | El Ordeño S. A. | Private sector |
| Guatemala | ASODEL (Dairy Development Association) | Private sector |
| Guatemala | Ministry of Agriculture, Livestock and Food | Government |
| Honduras | Programa Nacional de Alimentación Escolar | Government |
| Honduras | Cámara Hondureña de la Leche (CAHLE) | Private sector |
| Honduras | Federación Nacional de Agricultores y Ganaderos de Honduras (FENAGH) | Private sector |
| Honduras | Programa Nacional de Alimentación Escolar | Government |
| Latin America | Federación Panamericana de Lechería – Pan-American Dairy Federation (FEPALE) | NGO |

*Note: Names have been withheld to protect the informants’ anonymity.*
